# The Effects of Plant-Derived Phytochemical Compounds and Phytochemical-Rich Diets on Females with Polycystic Ovarian Syndrome: A Scoping Review of Clinical Trials

**DOI:** 10.3390/ijerph20156534

**Published:** 2023-08-06

**Authors:** Guadalupe Nayeli Chavez, Kataryna Jaworsky, Arpita Basu

**Affiliations:** 1 Department of Kinesiology and Nutrition Sciences, School of Integrated Health Sciences, University of Nevada, Las Vegas, NV 89154, USA; chaveg3@unlv.nevada.edu (G.N.C.); jawork1@unlv.nevada.edu (K.J.); 2 Kirk Kerkorian School of Medicine, University of Nevada, Las Vegas, NV 89106, USA

**Keywords:** polycystic ovarian syndrome, phytochemicals, antioxidants, hyperandrogenism, insulin resistance

## Abstract

Polycystic ovarian syndrome (PCOS) is an endocrine condition that impacts nutritional status, metabolic, and hormonal function in females of reproductive age. This condition is associated with increased androgen production (hyperandrogenism) and decreased insulin sensitivity, which often leads to insulin resistance and hyperinsulinemia. This increase in androgen production and insulin resistance is strongly associated with a high incidence of obesity, type-2 diabetes mellitus (T2DM), cardiovascular diseases (CVD), and certain types of gonad-related cancers among females who suffer from this condition. As research continues to grow, it has been demonstrated that PCOS is a complex condition, and some of its characteristics vary among the females that have this disorder. However, it has been suggested that oxidative stress and low-grade chronic inflammation could play an important role in the development of PCOS. Current evidence suggest that phytochemicals could potentially help with weight-loss by reducing oxidative stress and low-grade inflammation, as well as aid in metabolic and hormonal regulation due to their antioxidant properties. Some of the bioactive compounds found in plants that have shown positive effects in the attenuation of PCOS include flavonoids, polyphenols, phytoestrogen, and polyunsaturated fatty acids (PUFAs). Thus, a review of the current literature published on PCOS and phytochemicals was conducted in PubMed, Google Scholar, and the Academy of Nutrition and Dietetics databases for articles published between 2013 and 2023 with a study duration of 1 to 3 months and adequate sample sizes. The main purpose of this review of literature was to investigate the metabolic effects of phytochemical compounds and phytochemical-rich diets on females with PCOS by comparing the results of several randomized clinical trials.

## 1. Introduction

According to the Centers for Disease Control and Prevention (CDC), polycystic ovarian syndrome (PCOS) is one of the most common causes of female infertility, affecting about 6% to 12% of women of reproductive age in the United States [1]. Currently, there is limited data that specify which female ethnic groups are most affected by this condition. However, geographically speaking, a systematic review concluded that the prevalence of PCOS is higher in the southern region of the United States, with 47.5% of females being affected [2]. Another study conducted in India concluded that India and Iran were the countries with the highest incidence of PCOS cases, followed by Denmark and Brazil [3].

PCOS is an endocrine condition that impacts metabolic function of females in their reproductive years and occurs when a woman’s ovaries or adrenal glands produce more testosterone than normal [4]. In order to make a diagnosis of PCOS, there must be at least two out of three of the cardinal features of the Rotterdam criteria, which includes hyperandrogenism, oligo-anovulation, and polycystic ovaries visible on ultrasound, usually defined as greater than 12 follicles measuring 2–9 mm in diameter and/or an ovarian volume greater than 10 milliliters in at least one ovary [5]. PCOS often comes accompanied by additional physiological changes as well as physical changes. Some of the physiologic changes in PCOS include hyperandrogenism, hyperinsulinemia, insulin resistance, chronic anovulation, and impaired lipid metabolism [6]. Some of the physical changes in PCOS include acne, thinning scalp hair, as well as excess hair growth on the face and body, all due to the increase in testosterone production [7]. If left untreated, women who suffer from PCOS can develop serious health problems in the long run such as polycystic ovarian disease (PCOD)—the chronic form of PCOS—type-2 diabetes mellitus (T2DM), gestational diabetes, heart disease, high blood pressure, elevated low-density lipoprotein cholesterol (LDL-c), and decreased high-density lipoprotein cholesterol (HDL-c) blood serum levels [2]. Those who are overweight or obese are at an increased risk of developing these conditions.

**Figure 1:**
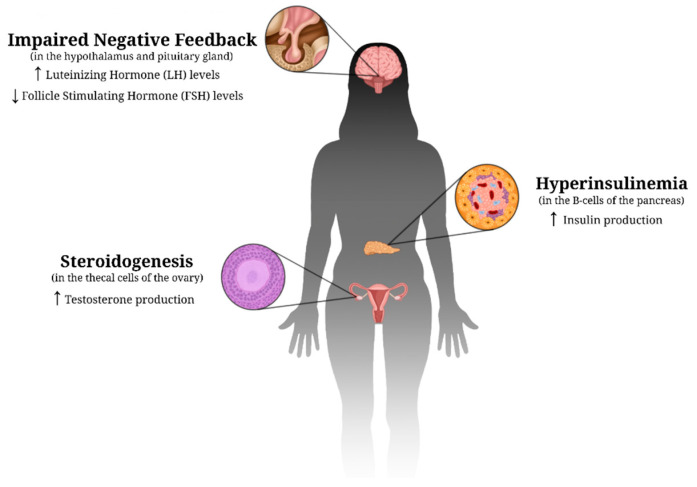
Proposed physiological changes in PCOS. These hormonal imbalances caused by impaired negative feedback lead to a decreased sensitivity to insulin. As a result, an environment is created in which the B-cells of the pancreas must secrete more insulin in order to compensate, leading to insulin resistance. Up (↑) and down (↓) arrows indicate an increase and decrease, respectively, in the specific hormone discussed.

Normally, estrogen and progesterone are the androgens that allow ovulation to occur in the female cycle, whereas testosterone is known as the “male androgen” and is responsible for masculine features [8]. As research continues to grow, it has been demonstrated that PCOS is a complex condition, and some of its characteristics vary among the females that suffer from it. As of today, the specific root cause of PCOS is still unknown, and it is thought to be a paradox [9]. However, what is known is that insulin plays an important role as it can stimulate steroidogenesis in the thecal cells of the ovaries, thus increasing androgen production. Insulin can also stimulate androgen secretion in the adrenal cortex to further increase the concentrations of androgens in the bloodstream [10]. Over time, this increase in androgens leads to decreased insulin sensitivity, which creates an environment where the body must secrete more insulin in order to compensate, leading to the development of insulin resistance. According to a recent systematic review study, evidence suggests that in women with PCOS, androgen excess has a detrimental impact on different metabolic tissues, including the adipose tissue (white and brown), liver, pancreas, and skeletal muscle [11]. The physiological effects of PCOS are illustrated in Figure 1.

PCOS and Oxidative Stress: Data suggest increased oxidative stress is present in females with PCOS. Oxidative stress refers to the imbalance of two opposite forces in the body, the production of ROS (reactive oxygen species) and antioxidants, in which the damaging effects of ROS are more powerful compared to the compensatory effect of antioxidants in the cells [12]. Two recent clinical trials noted that there is an increase in mitochondrial ROS production, as well as in the inflammatory enzyme myeloperoxidase (MPO), among females with PCOS, and the concentrations were even higher among those who presented with obesity and insulin resistance [13,14]. In more physiological implications, oxidative stress is thought to potentially lead to metabolic and hormonal imbalances, such conditions that are implicated in the development of PCOS [15].

PCOS and Chronic Inflammation: Data also suggest a strong association between low-grade chronic inflammation and an obesogenic state in females with PCOS, which is thought to be attributable to the accumulation of visceral fat. It was observed that women with PCOS often present with higher serum concentration of tumor necrosis factor (TNF) and C-reactive protein (CRP), as well as increased monocyte and lymphocyte circulating levels, and inflammatory infiltration in ovarian tissue [16,17]. A recent study indicated that concentrations of tumor necrosis factor alpha (TNF-α) and interleukin 6 (IL-6) are higher in females with PCOS, especially among those cases that present with signs of insulin resistance [13]. Other inflammatory markers that have shown to be permanently elevated in PCOS include IL-8 and IL-18 [18].

PCOS and Phytochemicals: Phytochemicals are chemical compounds found in vegetables, fruits, seeds, grains, herbs, spices, and plant-based beverages such as tea and wine. The frequent consumption of these compounds is associated with a decreased risk of several types of chronic inflammation related diseases due to their antioxidant and free radical scavenging effects [19]. It has been suggested that supplementation with natural compounds such as inositol, resveratrol, flavonoids, flavones, and omega-3 fatty acids may contribute to overcoming PCOS pathological features, including the presence of immature oocytes, insulin resistance, hyperandrogenism, oxidative stress, and low-grade chronic inflammation [20]. These antioxidant agents could potentially improve the homeostatic abnormalities that come with PCOS, reduce the risk of this syndrome, or aid with the management of the condition.

## 2. Methods

**Figure 2:**
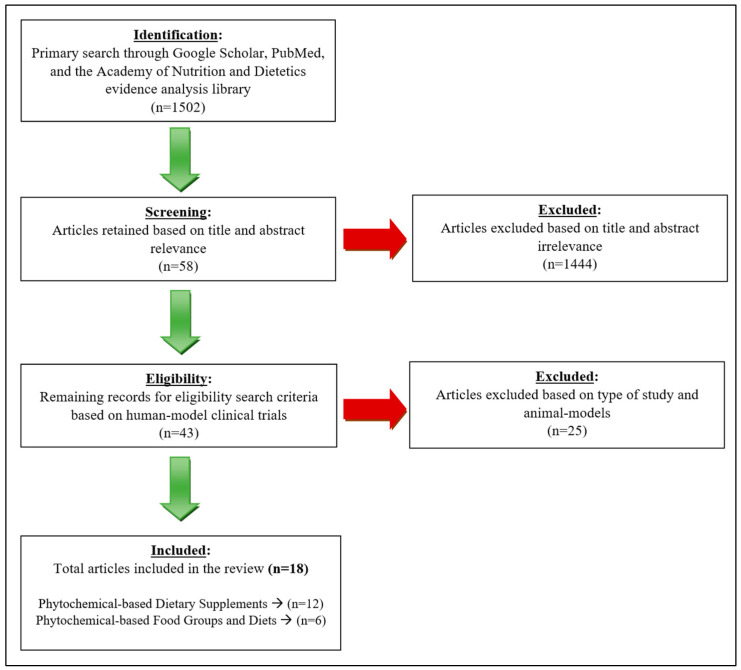
Search process of literature review.

The tools used to assess this literature review included the National Library of Medicine (PubMed), the UNLV library database, the Academy of Nutrition and Dietetics evidence analysis library, and Google Scholar. The search terms used to find the scientific articles included “phytochemicals and PCOS”, “phytonutrients and PCOS”, “antioxidants and PCOS”, “flavonoids and PCOS”, “flavanols and PCOS”, “anthocyanin and PCOS”, “EGCG and PCOS”, “curcumin and PCOS”, “quercetin and PCOS”, “resveratrol and PCOS”, “berberine and PCOS”, “plant-based diet and PCOS”, “Mediterranean diet and PCOS”, and “DASH diet and PCOS”. The criteria used to further narrow down the studies included: the year the studies were conducted (2013–2023), the length of study (1–3 months), sample size (>15 participants per study), and the search was limited to clinical trials. The search criteria is summarized in Figure 2.

### 2.1. Participants, Length of Studies, and Treatment

All of the clinical trials found were conducted on human participants. Some of the required physiologic characteristics for the selection criteria included at least three of the following: hyperandrogenism, anovulation, amenorrhea, insulin resistance, hirsutism, increased waist circumference, and/or unbalanced basic metabolic panel. The duration of the trials varied, and they ranged from 6 weeks to 12 weeks. The majority of the participants were middle-aged females, but the age groups ranged from 15 to 49 years old. All of the females included in the experimental groups of all clinical trials were overweight or obese. Diet designs and treatments varied in all clinical trials in terms of phytochemical compounds being studied and macronutrient arrangement.

### 2.2. Anthropometric Measurements

The tools used to assess the participants’ anthropometric measurements and body composition varied among all trials. However, some of the most remarkable measurements obtained included body weight, BMI (body mass index), waist circumference, and visceral fat volume.

### 2.3. Biochemical Data

Although biochemical data varied among all included trials, the most common biochemical values used to evaluate the metabolic and hormonal changes in the participants assessed for fasting androgens (testosterone, estradiol, progesterone, FSH, LH), fasting insulin levels, lipid profiles, fasting plasma blood glucose, glycosylated hemoglobin (HbA1c), as well as ovulatory function and menstrual cyclicity. The Homeostasis Model Assessment of Beta function (HOMA-β) was used to analyze beta cell function in the majority of the studies. Other biochemical compounds taken in consideration in a few studies included the sex hormone-binding globulin (SHBG), the sex hormone dehydroepiandrosterone sulfate (DHEAS), nitric oxide (NO), antioxidant capacity, AMP-activated protein kinase (AMPK), and adiponectin receptors (ADIPOR1 and ADIPOR2).

## 3. Results

In total, eighteen studies were included and analyzed in this review, which included seventeen randomized controlled trials (RCTs) and one matched clinical trial. Table 1 summarizes the significant findings of the twelve studies that focused on phytochemical-based supplement analysis in PCOS. 

Epigallocatechin-3-gallate, better known as EGCG, is a compound found in green tea. Three clinical trials that investigate the effects of EGCG on females with PCOS were found [21,22,23]. The length of the studies ranged from 45 days to 3 months. One 500 mg/day capsule of green tea extract (GTE) was prescribed to overweight and obese PCOS women in all studies. The age of the participants ranged from 18 to 55. One of the studies reported that body weight, BMI, waist circumference, and body fat percentage decreased statistically significantly after 45 days of treatment (*p* < 0.05) [22]. Another study showed a decrease in serum levels of fasting blood glucose levels, serum insulin levels, and free testosterone (*p* < 0.05) after 3 months of treatment [21]. The last and most recent EGCG study showed that most of the variables tested were significantly reduced—this reduction was statistically significant for weight (*p* < 0.001), BMI (*p* < 0.001), and waist circumference (*p* < 0.001) after 3 months of treatment [23].

Curcumin is a biologically active polyphenolic compound found in turmeric, a spice derived from the rhizomes of the plant *Curcuma longa*. Three clinical trials that investigated the effects of curcumin on healthy-weight females with PCOS were found [24,25,26]. The length of the studies ranged from 6 weeks to 3 months. Treatment consisted of 500 mg curcumin capsules. The age of the participants ranged from 18 to 49. One of the studies reported that serum insulin levels (*p* = 0.020) and Quantitative Insulin Sensitivity Check Index (*p* = 0.003) improved significantly after 6 weeks of treatment, while Homeostatic Model Assessment for Insulin Resistance (*p* = 0.067) improved marginally [26]. Another study showed that fasting blood glucose levels and dehydroepiandrosterone levels decreased significantly in the intervention group (*p* = 0.033), and there was a statistically non-significant increase (*p* = 0.082) in estradiol levels [25]. Lastly, the most recent study showed that curcumin significantly decreased weight (*p* = 0.03), BMI (*p* = 0.03), fasting glucose (*p* = 0.002), serum insulin (*p* = 0.02), insulin resistance (*p* = 0.02), total cholesterol (*p* = 0.001), LDL-cholesterol (*p* = 0.001), and total-/HDL-cholesterol ratio (*p* < 0.001). In addition, there was also a significant increase in HDL-cholesterol levels (*p* = 0.01) and insulin sensitivity (*p* = 0.02) [24].

Quercetin (flavonoid) is an antioxidant found naturally in red wine, apples, berries, and onions. Two clinical studies that investigated the effects of quercetin on overweight and obese females with PCOS were found [27,28]. The length of both studies was 3 months, and both studies were conducted in 2018. Treatment consisted of 1000 mg quercetin per day. The age of the participants ranged from 20 to 40. The first study showed that following supplementation, quercetin significantly decreased resistin hormone levels (*p* < 0.001), testosterone (*p* = 0.001), luteinizing hormone (*p* = 0.035), fasting blood glucose (*p* < 0.001), insulin (*p* = 0.02), as well as Homeostatic Model Assessment of Insulin Resistance (*p* = 0.009) [27]. The second study focused on investigating the effects of quercetin on ADIPOR1 and ADIPOR2 receptors, which regulate glucose and fatty acid metabolism partly via the activation of AMP-activated protein kinase in the adiponectin signaling pathway. The study indicated that oral quercetin supplementation significantly increased ADIPOR1 and ADIPOR2 transcript expression (*p* < 0.01), and it also enhanced AMP-activated protein kinase levels (*p* < 0.05) [28].

Resveratrol is a polyphenol, a naturally occurring highly powerful antioxidant, commonly found in blueberries, grapes and its product, wine. Two clinical studies that investigated the effects of resveratrol on overweight women with PCOS were found [29,30]. The length of both studies was 3 months. The quantity of resveratrol given to the participants varied between the two studies at 1000–1500 mg resveratrol per day. The age of the participants ranged from 18 to 40. The first study demonstrated a significant decrease in total testosterone levels by 23.1% (*p* = 0.01), a 22.2% decrease of dehydroepiandrosterone sulfate (*p* = 0.01), a decrease in fasting insulin levels by 31.8% (*p* = 0.007), and an increase in insulin sensitivity index by 66.3% (*p* = 0.04). However, gonadotropin levels, lipid profiles, inflammation markers, and endothelial function were not significantly altered [29]. The second and more recent study showed that women who received resveratrol had a statistically higher regular menstruation rate (*p* = 0.03) and lower hair loss (*p* = 0.009). No significant changes were noted on ovarian and adrenal androgens, sex hormone-binding globulin levels, free androgen index, or lipid profiles [30].

Berberine is a chemical compound and antioxidant found in barberries. Two clinical studies that investigated the effects of berberine on obese and overweight females with PCOS were found [31,32]. The length of the studies ranged from 3 months to 6 months. Treatment plans varied between the two studies. The age of the participants was not specified in either of the two studies. The first study showed a significant improvement in total testosterone (*p* < 0.01), free androgen index (*p* < 0.01), androstenedione (*p* < 0.01), sex hormone-binding globulin (*p* < 0.01), progesterone (*p* < 0.01), total cholesterol (*p* = 0.01), low density lipoprotein cholesterol (*p* < 0.01), triglycerides (*p* < 0.01), menses frequency (*p* < 0.01), and waist circumference (*p* = 0.04) after 6 months of treatment, in which PCOS women received 500 mg berberine twice times daily [31]. The second study showed slight reductions in total testosterone, free androgen index, fasting glucose, fasting insulin, and homeostatic model assessment for insulin resistance, as well as increased the pregnancy rate before in vitro fertilization after taking three 500 mg berberine tablets per day for 3 months (*p* < 0.05) [32]. 

Table 2 summarizes the significant findings of the six studies that were analyzed for their results on phytochemical-based food groups and diets in PCOS. 

Pomegranate juice gained popularity due to its antioxidant and anti-inflammatory effects. Two clinical trials investigating the metabolic effects of pomegranate juice on females with PCOS were found [33,34]. The duration of both studies was 8 weeks. In one study, the treatment consisted of 2 L of pomegranate juice per week on females aged 15 to 48 years old. In comparison with the baseline, BMI, weight, and waist circumferences decreased after 8 weeks of treatment (*p* < 0.05), the changes in fasting blood glucose were significant (*p* < 0.05), serum insulin levels decreased (*p* < 0.05), insulin sensitivity increased (*p* < 0.05), and testosterone was reduced significantly (*p* < 0.05) [33]. The other study’s treatment consisted of 45 milliliters/day of concentrated pomegranate juice in obese/overweight females aged 18–40 years old. The results indicated that serum testosterone levels were significantly decreased (−0.004 ± 0.013 vs. 0.039 ± 0.013, *p* = 0.039) when compared with control groups [34].

When investigating the effects of phytochemical-rich diets on PCOS, four RCTs were found- three implemented the DASH (Dietary Approaches to Stop Hypertension) dietary pattern and one implemented the Mediterranean diet (MED-diet) [35,36,37,38]. In the studies following the DASH diet, the prescribed dietary arrangement consisted of 50–55% carbs, 15–20% protein, and 25–30% total fat in all studies. One of the studies showed a significant reduction in serum insulin levels (*p* = 0.03), HOMA-IR score (*p* = 0.01), serum CRP levels (*p* = 0.009), as well as a significant reduction in waist (*p* = 0.003) and hip circumference (*p* < 0.001) after 8 weeks of treatment in overweight and obese women [35]. A longer study conducted in obese/overweight females aged 20–40 years old indicated that the consumption of a DASH diet compared to a control diet was associated with a significant reduction in weight (*p* = 0.032), body mass index (*p*= 0.02), fat mass (*p* = 0.008), serum androstenedione (*p* = 0.019), and increased concentrations of SHBG (*p* = 0.003) after 3 months of treatment [36]. A more recent study also conducted on overweight and obese women aged 18–40 years old concluded that adherence to the DASH diet results in a significant decrease in BMI (*p* = 0.02). Significant decreases in HOMA-IR (*p* = 0.02), free androgen index (*p* = 0.02), and malondialdehyde (MDA) levels (*p* < 0.001), as well as significant increases in Quantitative Insulin Sensitivity Check Index (*p* = 0.02), SHBG (*p* = 0.01), and nitric oxide (*p* < 0.001) after 12 weeks of treatment [37]. Lastly, the Mediterranean (MED-diet) model was conducted on obese/overweight females aged 16–45 years old for 12 weeks. In this study, the results indicated a significant reduction trend in weight (*p* < 0.05), BMI (*p* < 0.05), waist circumference (*p* < 0.05), total testosterone (*p* < 0.001), LH (*p* < 0.05), LH/FSH (*p* < 0.05), and fasting plasma glucose (*p* < 0.001). Improved HOMA-IR index (*p* < 0.05), QUICKI (Quantitative Insulin Sensitivity Check Index) index (*p* < 0.05), triglycerides (*p* < 0.05), total cholesterol (*p* < 0.05), and LDL-c (*p* < 0.05) were also observed [38]. 

## 4. Discussion

PCOS and PCOD are metabolic conditions that necessitate a multifocal approach to management of the sequelae of the disease, including both lifestyle interventions along with pharmacologic treatments. One of the mainstays of pharmacologic treatment in PCOS are anti-androgen medications, which can augment weight loss and improvement of hyperandrogenic-related symptoms. However, there are limitations in the use of these anti-androgen medications, especially in patients who are pregnant or hoping to become pregnant as there may be an increased risk of feminization of a male fetus, thus necessitating other therapeutic options that can achieve similar anti-androgenic effects [39]. In clinical practice, the lifestyle interventions recommended for management of PCOS revolve around weight loss given that part of the disease pathogenesis of PCOS is due to excess adipose tissue [40]. While overall weight loss is important, there is a new focus on specific dietary supplementation with various antioxidants and flavonoids that can be beneficial in PCOS by both increasing weight loss as well as modulating various biochemical and metabolic markers of PCOS disease severity, and cohesive resources that summarize the available supplements are lacking. Thus, the goal of this review was to examine and summarize the effects of supplementation with plant-based phytochemicals on the multiple markers of disease severity in PCOS.

### 4.1. Phytochemical-Based Supplements and PCOS

The twelve studies examined for the effects of phytochemical-based supplementation on markers of PCOS, including daily supplementation of either EGCG, curcumin, quercetin, resveratrol, or berberine, all resulted in significant improvements of various biochemical markers of PCOS (Table 1) [21,22,23,24,25,26,27,28,29,30,31,32]. EGCG, a phytochemical found in high concentrations in green teas, resulted in significant reductions in weight, BMI, waist circumference, and body fat, decreased levels in fasting insulin and fasting glucose levels, and decreased free testosterone levels [21,22,23]. It has been demonstrated that insulin resistance, one of the key aspects in the pathogenesis in both PCOS and its side effects, is directly due to the overexposure of androgens in patients with PCOS [6]. Thus, we can infer that decreased levels of androgens, including testosterone, should result in improved insulin sensitivity in these patients. Our review showed that EGCG directly modulates both androgen levels via decreasing testosterone as well as insulin sensitivity via decreased fasting insulin levels and improved HOMA-IR scores, which, in turn, results in lower fasting glucose levels via enhanced glucose uptake by insulin-regulated glucose transporters such as GLUT-4 [41]. Furthermore, our findings align with those elucidated by Kamal et al., 2021 in their review on green tea catechins, including EGCG, and their impact on PCOS, which demonstrated that increased green tea in the diet of females with PCOS resulted in enhanced ovulation, reduced cyst formation, and improved hyperalgesia [42]. These effects, including improved ovulation in PCOS patients who otherwise often experience anovulation, are likely secondary to the decreased levels of androgens circulating in the body as a result of EGCG. Although the exact mechanism of EGCG and its implications in various metabolic disorders including PCOS have yet to be elucidated, it has been well documented that EGCG has anti-inflammatory and antioxidant effects [43]. This review adds to the literature that demonstrates these beneficial effects on EGCG specifically on PCOS management via decreased anthropometric and metabolic markers of PCOS and should be considered when formulating a treatment plan in PCOS patients.

Curcumin, a biologically active polyphenolic compound found in turmeric, supplementation was examined by three RCTs and was found to be effective in reducing weight, BMI, fasting glucose, serum insulin, total cholesterol, and LDL-cholesterol [24,25,26]. Our findings mirror those found in the review by Venkatesan et al., 2022, which demonstrated that supplementation of curcumin alone in the diets of PCOS patients resulted in improved glycemic and lipid profiles as well as reduced BMI [44]. Curcumin has been demonstrated to activate various insulin receptors as well as inhibit the production of numerous pro-inflammatory cytokines that are responsible for the pathogenesis of metabolic disorders [45], specifically in type 2 diabetes. The insulin resistance in PCOS has a similar pathogenesis to that in type 2 diabetes; thus, curcumin likely exerts similar effects in the insulin resistance pathway in PCOS patients. Furthermore, Venkatesan et al.’s review was able to demonstrate a significant improvement in circulating androgen levels associated with PCOS [44]. It has been documented that the excessive androgens present in PCOS result in endoplasmic reticulum stress in ovarian granulosa cells, resulting in proliferation of follicles via the phosphatidylinositol-3-kinase (PI3K/AKT) pathway and ultimate formation of the ovarian cysts seen in PCOS [46]. While the RCTs included in our review that examined curcumin did not result in significant changes in androgen levels, it has been elucidated that curcumin protects ovarian granulosa cells from this oxidative stress that results from hyperandrogenism. Thus, supplementation of curcumin in PCOS patients is beneficial in numerous ways, both in decreasing insulin resistance and improving insulin and glucose levels as well as exerting a protective effect against the hyperandrogenic effects in PCOS.

Supplementation with quercetin, a flavonoid found in foods such as berries, wine, and onions, resulted in improved fasting blood glucose and insulin, improved insulin resistance, decreased resistin plasma levels, decreased testosterone and LH concentrations, and increased adiponectin receptor and AMP-K levels as demonstrated by two RCTs [27,28]. Importantly, a high LH level, more specifically a high LH:FSH ratio, is pathognomonic for PCOS and is implicated in the anovulation that many patients with PCOS experience due to the lack of a normal LH surge [47]. Our review highlighted that quercetin supplementation results in decreased LH concentrations along with decreased testosterone concentrations, which are both implicated in menstrual regularity and the hormonal side effects of PCOS. These findings are in agreement with the results of the review by Pourteymour Fard Tabrizi et al., 2020, who similarly demonstrated reduced testosterone, LH, and insulin resistance with quercetin supplementation [48]. While the exact mechanisms of quercetin’s effects on the hormonal pathways in PCOS patients are still being examined, it was postulated by Ma et al., 2022, that increased levels of quercetin result in downregulation of the C-type natriuretic peptide/natriuretic peptide receptor 2 (CNP/NPR2) pathway, a pathway that normally results in blocked oocyte meiosis and altered ovulation when upregulated, which restores ovulatory normalcy and alleviates the menstrual symptoms of PCOS [49]. Thus, quercetin supplementation is another possible avenue of dietary modifications that are beneficial in PCOS management.

Resveratrol supplementation, a polyphenol with highly antioxidant properties, was found to result in a significant reduction in ovarian and adrenal androgens, testosterone, and DHEA, as well as improve menstrual cycle regularity and hair loss [29,30]. There is a paucity of studies regarding resveratrol supplementation in PCOS and, similarly, a lack of reviews discussing the overall effects of resveratrol in PCOS pathogenesis. Resveratrol supplementation has been demonstrated in rat models to restore the thickness and number of granulosa cells in the ovaries, decrease body weight, lower testosterone levels, and restore the glycolytic process by modulated expression of various rate limiting enzymes of glycolysis [50]. Although this mechanistic research was conducted in rat models, it can be inferred that the pathogenesis of PCOS in rat models and humans is similar and, thus, that resveratrol supplementation would have similar effects in humans, as demonstrated by the results of our review. Thus, the beneficial effects seen with resveratrol supplementation in humans conducted in the RCTs in our review likely were due to the same upregulation of SIRT2, a transcription factor that regulates the acetylation of enzymes involved in ovarian glycolysis, as well as improved energy metabolism in the ovaries, which is protective against PCOS [50]. The results of our review, specifically that resveratrol supplementation resulted in improved menstrual cycle regularity and reduction in ovarian androgens, concur with the findings of Liang et al.’s review, in 2021, of the mechanism of resveratrol in PCOS as well as the results of the review by Shojaei-Zarghani et al., 2022, who similarly demonstrated the benefits of resveratrol supplementation on improved hormonal profiles in PCOS patients [50,51].

Finally, increasing berberine levels in the diet resulted in improved levels of testosterone, androstenedione, sex hormone-binding globulin, and progesterone, as well as total cholesterol, LDL-cholesterol, and triglycerides [31,32]. These positive effects of berberine supplementation in PCOS patients were similarly demonstrated in the randomized controlled trial by Mishra et al., 2022, which compared the effects of berberine supplementation to treatment with metformin, one of the mainstay treatments of PCOS currently [52]. They were able to demonstrate that supplementation of berberine alone showed greater differences in weight, BMI, fasting blood sugar, total testosterone, free androgen index, total cholesterol, LDL, and HDL cholesterol as compared to both the metformin and myoinositol groups [52], highlighting that supplementation with phytochemicals easily found in diets may be outperforming the traditional pharmacologic measures used to treat PCOS. Furthermore, one study included in our review even reported improved menses frequency in the PCOS patients who supplemented additional berberine in their diet [32]. As with many of the phytochemicals discussed previously, the majority of the mechanistic research into berberine and PCOS was carried out in rat models and, as such, studies on human participants are lacking. The experiments conducted by Wang et al., 2021, demonstrated that PCOS rats supplemented with berberine had decreased levels of serum LH and total cholesterol, improved glucose tolerance, and overall improved regularity of menses [53]. They postulated that the mechanism of improved menstrual regularity is associated with the upregulation of the luteinizing hormone/choriogonadotropin receptor (LHCGR) and cytochrome P450 Family 19 Subfamily A Member 1 (CYP19A1) in the granulosa cell of ovaries, which was directly upregulated with berberine supplementation [53]. Therefore, berberine supplementation has been demonstrated to improve metabolic, hormonal, and clinical outcomes in PCOS patients and should be considered in the PCOS treatment regimen.

Overall, our review compiled numerous RCTs and clinical trials that demonstrate supplementation of the diets of PCOS patients with sources rich in EGCG, curcumin, quercetin, resveratrol, and berberine results in various improvements in anthropometric, metabolic, and hormonal markers that are all implicated in PCOS. Based on the results of the various articles included, we can postulate that supplementation of the diet with similar dosages as administered in the cited studies, such as 500 mg of green tea extract daily or 1000 mg of quercetin daily, can be beneficial in controlling the sequelae of PCOS, as they showed significance with daily intake. The route of administration, whether it be via capsules containing these beneficial phytochemicals or direct supplementation via dietary sources, is dependent upon the ease of access and usability, which is unique to every patient and should be discussed with the healthcare provider if these phytochemicals are implemented in the treatment plan of PCOS. Thus, supplementation with dietary sources rich in these phytochemicals, including green tea and berries, is beneficial in improving hormonal regularity in patients with PCOS and ultimately improving both the symptoms and sequalae of PCOD.

### 4.2. Phytochemical-Based Food Groups/Diets and PCOS

Six RCTs examined the influence of specific phytochemical-rich foods and diet regimens, including pomegranate juice, DASH diets, and the Mediterranean diet, on the various anthropometric, metabolic, and hormonal parameters measured in PCOS patients (Table 2) [33,34,35,36,37,38]. Dietary supplementation with pomegranate juice resulted in improved insulin resistance, decreased testosterone levels, improved BMI and overall weight, as well as increased total antioxidant levels [33,34]. Pomegranate is a rich source of anthocyanins, which is part of a specific group of phytochemicals called flavonoids, which have potent antioxidant and anti-inflammatory effects in humans [54]. While the exact mechanism of pomegranate supplementation and its effect in the various metabolic and hormonal markers of PCOS have yet to be elucidated, the results of our review demonstrate the beneficial effects of pomegranate in PCOS patients. These results mirror the results found in the experiment conducted by Ibrahim et al., 2022, who demonstrated that supplementation of pomegranate extract in rats with PCOS resulted in improved testosterone levels as well as reduced endometrial oxidative stress that contributes to hyperalgesia in PCOS and normalization of androgen receptor expression [55]. Furthermore, the review on pomegranate and its effects in metabolic syndrome by Medjakovic et al., 2013, highlighted the beneficial effects of pomegranate in its inhibition of alpha-glucosidase and upregulation of glucose transporter type 4 (GLUT4), both of which increase insulin sensitivity and improve glycemic control [56]. PCOS is another type of metabolic syndrome, especially as it often comes with the sequalae of type 2 diabetes and other signs of metabolic dysfunction, which benefits from the supplementation of pomegranate juice. Thus, pomegranate supplementation is a potential additional supplementation method that can be used in the management of PCOS given its metabolic and hormonal normalization.

Three RCTs examined the implementation of the DASH diet in PCOS patients and demonstrated a reduction in serum insulin levels, improved HOMA-IR scores, decreased CRP levels, improved SHBG capacity, reduced androstenedione levels, and reduction in various anthropometric measures of weight [35,36,37]. The DASH diet is an anti-inflammatory diet characterized by a diet low in salt and rich in fruits, vegetables, whole grains, low-fat dairy, and lean proteins [57]. The systematic review conducted by Shang et al., 2020, examined various dietary regimens in the management of PCOS and found that the DASH diet was the most effective in improving insulin sensitivity in PCOS, mirroring the results of our review that showed improved HOMA-IR (a measurement tool that approximates insulin resistance) [58]. Few studies besides the RCTs included in our trial investigated the DASH diet and its effect on the various hormones implicated in PCOS, highlighting the need for more research into the dietary modulation of hormones in PCOS. The RCT conducted by Daneshzad et al., 2022, demonstrated that the implementation of the DASH diet in women with type 2 diabetes resulted in decreased testosterone and fasting blood sugars [59]. Furthermore, Liu et al., 2022, demonstrated that pro-inflammatory diets, as measured by the dietary inflammatory index, resulted in decreased sex hormone-binding globulin [60], which is a biomarker that is pathognomonic for PCOS as low levels of SBHG result in hyperandrogenism and insulin resistance [61]. While these studies were not conducted in patients with PCOS, the results support the findings of our review in that an anti-inflammatory diet such as the DASH diet results in improved metabolic markers of insulin resistance, one of the key pathways implicated in PCOS, as well as improved levels of SHBG, thus lowering hyperandrogenism and insulin resistance seen in PCOS. Therefore, the DASH diet can be recommended clinically for patients with PCOS to regulate both the hormonal and metabolic derangements seen in PCOS.

Finally, one RCT examined the effects of the Mediterranean diet on various outcomes of PCOS and demonstrated improved overall menstruation rates, decreased total testosterone levels, decreased LH levels, improved anthropometric measures of weight, improvement of various lipid parameters including decreased triglycerides, total cholesterol, and LDL cholesterol, reduction in blood glucose, and improved HOMA-IR levels [38]. The Mediterranean diet, characterized as a diet rich in complex carbohydrates, fiber, and monosaturated fats, has been well documented in its anti-inflammatory and antioxidant capabilities and its subsequent improvement in lipid profiles, insulin sensitivity, and endothelial function [62]. It has been documented that adherence to a Mediterranean diet is associated with improvements in ovarian form via the modulation of obesity, insulin resistance, and hyperandrogenism [63], paralleling the results of our review that demonstrate decreased androgen levels and improvement of various metabolic markers in PCOS. Additionally, the case–control, cross-sectional study conducted by Barrea et al., 2019, demonstrated a direct association between the adherence of a Mediterranean diet and the clinical severity of PCOS in patients, exhibited by increased testosterone, elevated CRP, and increased measures of body composition [64]. These findings complement the findings of our review in that the Mediterranean diet is beneficial in controlling both the hormonal and metabolic effects of PCOS in females.

Overall, this review demonstrated that various dietary pattern changes, such as the DASH diet and Mediterranean diet, are beneficial in the management of PCOS via improvement in metabolic factors such as glucose and insulin as well as normalization of various hormonal components of PCOS, including testosterone and LH. Specifically, the addition of two liters of pomegranate juice, following the DASH diet recommendations of 50–55% carbohydrates, 15–20% protein, and 25–30% total fat, and the Mediterranean diet with low carbohydrate intake are ways that patients can use their dietary habits to help control the various symptoms that they may experience with PCOS. Therefore, these dietary patterns should be included in the management of PCOS.

### 4.3. Strengths and Limitations

This review of phytochemical-based supplements and dietary changes on the various metabolic, hormonal, and clinical outcomes of PCOS had numerous strengths. First, this review offers a comprehensive examination of the published studies that investigated phytochemicals and their role in the pathogenesis and management of PCOS, an area of research that is unfortunately lacking. Secondly, there is a wide variety of phytochemical supplements and dietary models that were discussed in this review. The results of twelve clinical trials that examined five unique phytochemicals (EGCG, curcumin, quercetin, resveratrol, and berberine) were discussed, providing a wide variety of potential supplements that can be used clinically. Furthermore, phytochemical-rich dietary patterns were also examined in this review, including the DASH diet and Mediterranean diet, which allow for a whole dietary plan to be constructed for a patient with PCOS. Ultimately, the phytochemicals discussed in this review are cost-effective and readily available to patients, as they are often found in typical grocery store items such as green tea, turmeric, and berries, as compared to the standard pharmacologic tools used in PCOS management. Specific recommendations for use of phytochemical and dietary interventions to help manage PCOS are unique to the patient, as each patient has varying access to certain phytochemicals, personal preference with taking capsules versus obtaining the recommended intake directly from the diet, and various other factors that must be discussed between the patient and their healthcare providers. Ultimately, any of the options discussed in the included 18 clinical trials are options that patients can use to help augment their treatment of PCOS. Thirdly, all studies included in this review article were randomized controlled trials and matched clinical trials, all of which had a control or placebo group, which allowed the authors of each individual study to determine causality between their specific phytochemical intervention and the PCOS outcome measures, and, in turn, allows us to assume that the benefits seen in the patients across all studies are due to those specific phytochemical supplementations. Finally, this review offers clear summative suggestions for phytochemical-based supplementation and dietary modifications for glycemic, hormonal, and clinical outcomes in patients with PCOS.

However, we acknowledge the limitations present within our review as well. First, the studies included in this review were limited by their study duration to 1 to 3 months. Although this is an adequate time period for evaluating many of the markers of PCOS management, especially those related to the metabolic parameters, many of the hormonal outcomes of PCOS, specifically regarding menstrual regularity and hormonal regulation, often take many months to regulate. Furthermore, a majority of the randomized controlled trials included in this review were conducted in non-Western countries, mainly in Iran and China. These countries have different dietary habits and nutritional intakes as compared to the United States or other European countries and, thus, it is important to note that these different baseline intakes may affect the overall significance of these supplements in the diet. Finally, we did not perform a quantitative meta-analysis of the reported small number of trials for each supplement and dietary pattern, and this must be addressed in future investigation. Despite these limitations, we believe that the results of this review fill an important knowledge and research gap by providing a summative resource of various phytochemical-based supplementations and dietary changes that can be made in order to improve the various metabolic, hormonal, and clinical outcomes important to patients with PCOS.

## 5. Conclusions

In conclusion, this literature review consisted of 18 randomized controlled and clinical trials investigating the effects of phytochemical-rich supplements, food groups, and diets on females with PCOS, demonstrating that the addition of phytochemicals into the diet have significant antioxidant and anti-inflammatory properties, which result in improved hormonal and metabolic markers in women with PCOS. Future directions include the investigation of more phytochemical groups with a closer approach to antioxidant and anti-inflammatory markers. Therefore, supplementation with phytochemical compounds can potentially be seen as a complementary practice that can be combined with medical nutrition therapy and lifestyle modifications in the management of PCOS outcomes.

## Figures and Tables

**Table 1:** Effects of phytochemical-based supplements on females with PCOS (Clinical Trials).

Phytochemical Compound	Author/Year	PCOS Duration, Sample Size, and Treatment Plan	Significant Findings (*p* < 0.05)
EGCG	(Tehrani, 2017) [21]	3 months Overweight and obese women 20–40 years old (*n* = 60) Tx: One 500 mg green tea tablet per day	weight reduction decrease in fasting insulin decrease in free testosterone levels
	(Mombaini, 2017) [22]	45 days Women aged 18–55 years with BMI 20–35 kg/m^2^ (*n* = 45) Tx: One 500 mg green tea leaf powder tablet per day	significant reduction in weight, BMI, WC, and body fat
	(Farhadian, 2020) [23]	3 months Overweight women 18–35 years old (*n* = 15) Tx: One 500 mg green tea leaf powder tablet per day	significant reduction in anthropometric indices such as weight, BMI, and waist and hip circumference
Curcumin	(Jamilian, 2020) [24]	3 months Women aged 18–40 (*n* = 60) Tx: 500 mg/day curcumin	significant weight and BMI reduction decrease in fasting glucose decrease in serum insulin improved HOMA-IR (insulin resistance) decrease in total cholesterol and LDL-c
	(Heshmati, 2020) [25]	3 months Women aged 18–49 (*n* = 67) Tx: 500 mg curcumin powder in a capsule 3 times/day	Decreased fasting plasma glucose (FPG)
	(Sohaei, 2019) [26]	6 weeks Women aged 18–40 years old (*n* = 27) Tx: 500 mg curcumin 2 times/day	Quantitative Insulin Sensitivity Check Index (QUICKI) improved significantly Homeostatic Model Assessment for Insulin Resistance (HOMA-IR) improved marginally
Quercetin	(Khorshidi, 2018) [27]	3 months Women with BMI 25–40 kg/m^2^, aged 20–40 (*n* = 27) Tx: 1000 mg quercetin per day	Decreased resistin plasma levels and gene expression Decreased testosterone and LH concentrations FBG, fasting insulin, and insulin resistance were improved significantly
	(Rezvan, 2018) [28]	3 months Overweight or obese women, mean age 29 (*n* = 42) Tx: Two 500 mg capsules of quercetin daily	Increased adiponectin receptors (ADIPOR1 and ADIPOR2) Enhanced AMPK levels
Resveratrol	(Banaszewska, 2016) [29]	3 months Overweight women (*n* = 15) Tx: micronized transresveratrol; 1500 mg/day	significant reduction in ovarian and adrenal androgens, testosterone and DHEAS.
	(Mansour, 2021) [30]	3 months Women aged 18–40 (*n* = 39) Tx: 1000 mg resveratrol/day	improved menstrual cyclicity and hair loss.
Berberine	(Orio, 2013) [31]	6 months Obese women (*n* = 50) Tx: 588 mg *Berberis aristate* and 105 mg of *Silybum marianum*—1 tablet, 2 times/day	improved HOMA-IR and hormonal profiles
	(An, 2014) [32]	3 months Overweight women (*n* = 41) Tx: Berberine tablets 500 mg, 3 times/day	improved metabolic profile improved HOMA-IR improved respondence to ovarian stimulation

Abbreviations: AMPK: AMP-activated protein kinase; BMI: body mass index; DHEAS: dehydroepiandrosterone sulfate; EGCG: epigallocatechin-3-gallate; FBG: fasting blood glucose; FPG: fasting plasma glucose; HOMA-IR: homeostatic model assessment for insulin resistance; kg/m^2^: kilograms/square meter; LDL-c: low density lipoprotein cholesterol; LH: luteinizing hormone; mg: milligrams; *n*: sample size; PCOS: polycystic ovarian syndrome; QUICKI: quantitative insulin sensitivity check index; Tx: treatment; WC: waist circumference.

**Table 2:** Effects of Phytochemical-based Food Groups and Diets on Females with PCOS (Clinical Trials).

Food Groups	Author/Year	PCOS Duration, Sample Size, and Treatment Plan	Significant Findings (*p* < 0.05)
*Pomegranate juice*	(Esmaeilinezhad, 2019) [33]	8 weeks PCOS women aged 15–48 (*n* = 23) Tx: 2 L of pomegranate juice per week	improved insulin resistance improved serum insulin level improved testosterone levels improved BMI, weight and waist circumference
	(Abedini, 2023) [34]	8 weeks Overweight and obese women with PCOS aged 18–40 (*n* = 21) Tx: 45 mL of concentrated pomegranate juice + 180 mL H_2_O per day	Improved serum testosterone levels
**Diets**	**Author/Year**	**PCOS Duration, Sample Size,** **and Treatment Plan**	**Significant Findings (*p* < 0.05)**
*DASH diet*	(Asemi, 2015) [35]	8 weeks Overweight and obese women with PCOS (*n* = 23) Tx: DASH diet (52% carbs, 18% protein, and 30% total fat)	reduction in serum insulin levels improved HOMA-IR score improved serum hs-CRP levels reduction in waist and hip circumference
	(Azadi-Yazdi, 2017) [36]	3 months Obese and overweight women with PCOS aged 20–40 (*n* = 28) Tx: DASH diet (50–55% carbs, 15–20% protein, and 25–30% total fat)	decreased fat mass reduced androstenedione levels improved SHBG capacity improved total antioxidant capacity reduced serum testosterone levels
	(Foroozanfard, 2017) [37]	3 months Overweight and obese women with PCOS (*n* = 30) Tx: Low calorie DASH diet (52–55% carbs, 16–18% protein, and 30% total fat)	weight and BMI reduction reduction in serum insulin levels improved HOMA-IR score improved HOMA-B score improved serum SHBG levels and NO levels
*Mediterranean Diet*	(Mei, 2022) [38]	3 months Overweight women (*n* = 30) Tx: low carb (<20%) MED diet	improved menstruation improved BMI, weight and waist circumference decreased total testosterone levels decreased LH levels improved HOMA-IR index improved QUICKI index improved lipid parameters: TG, TC, and LDL-C decreased significantly reduction in blood glucose

Abbreviations: BMI: body mass index; DASH: Dietary Approaches to Stop Hypertension; H_2_O: water; HOMA-B: homeostatic model assessment of beta function; HOMA-IR: homeostatic model assessment for insulin resistance; hs-CRP: highly sensitive C-reactive protein; kg/m^2^: kilograms/square meter; L: liters; LDL-c: low density lipoprotein cholesterol; LH: luteinizing hormone; MED: Mediterranean; mL: milliliter; *n*: sample size; NO: nitric oxide; PCOS: polycystic ovarian syndrome; QUICKI: quantitative insulin sensitivity check index; SHBG: sex hormone-binding globulin; TC: total cholesterol; TG: triglycerides; Tx: treatment.

## Data Availability

Not applicable.
